# Constitutive and Operational Variation of Learning in Foraging Predatory Mites

**DOI:** 10.1371/journal.pone.0166334

**Published:** 2016-11-04

**Authors:** Michael Seiter, Peter Schausberger

**Affiliations:** 1 Group of Arthropod Ecology and Behavior, Department of Crop Sciences, University of Natural Resources and Life Sciences, Vienna, Austria; 2 Department of Behavioural Biology, University of Vienna, Vienna, Austria; Chinese Academy of Agricultural Sciences Institute of Plant Protection, CHINA

## Abstract

Learning is widely documented across animal taxa but studies stringently scrutinizing the causes of constitutive or operational variation of learning among populations and individuals are scarce. The ability to learn is genetically determined and subject to constitutive variation while the performance in learning depends on the immediate circumstances and is subject to operational variation. We assessed variation in learning ability and performance of plant-inhabiting predatory mites, *Amblyseius swirskii*, caused by population origin, rearing diet, and type of experience. Using an early learning foraging paradigm, we determined that homogeneous single prey environments did not select for reduced learning ability, as compared to natural prey-diverse environments, whereas a multi-generational pollen diet resulted in loss of learning, as compared to a diet of live prey. Associative learning produced stronger effects than non-associative learning but both types of experience produced persistent memory. Our study represents a key example of environmentally caused variation in learning ability and performance.

## Introduction

Learning definitions differ between scientific disciplines and contexts [1], but from a behavioral perspective learning is broadly defined as experience-based change in behavior [2]. Learning is an omnipresent phenomenon in animals and well known from both invertebrates and vertebrates [2–5]. Among arthropods, learning is especially well-studied in social hymenopterans such as ants and bees [6,7] and model animals such as fruit flies *Drosophila* sp. [8,9]. Learning is a highly important tool for animals to flexibly adjust their behavior in variable environments. While learning as such is well documented across animal taxa, rigorous studies addressing the factors causing variation of learning are scarce [10]. The ability to learn and to memorize past experiences is genetically determined, shaped by natural selection and, thus, subject to constitutive variation; the learning performance is strongly dependent on the immediate context and, thus, subject to operational variation. Here, we scrutinized constitutive and proximate (operational) variation in learning by the plant-inhabiting predatory mite *Amblyseius swirskii* Athias-Henriot (Phytoseiidae) in foraging contexts. We compared the learning ability of two different populations (commercially mass-reared versus natural free-living) and examined the influence of diet history (plant versus animal diet) and type of learning (associative versus non-associative) on learning performance.

Cognitive abilities including learning and memory vary among individuals, populations and species, in dependence of their environments [10–12]. Different populations of a species often live in different environments, which pose varying selection pressures on the individuals of those populations and can thus select for different learning and memory abilities [4,13,14]. For example, many parasitoid wasps are well able to learn which cues are best suited for host location. These cues vary with the environment and can be unpredictable in complex fluctuating habitats. Therefore, the ability to learn allows the wasps to selectively focus on those cues that reliably guide them to their hosts. In contrast, in a stable, predictable, homogeneous habitat, that is, with no or only little environmental fluctuations, the learning abilities used for host finding can be strongly reduced or even absent [13]. For example, Brydges *et al*. [14] showed for three-spined sticklebacks that specimens from a stable habitat, a pond, had a shorter memory duration after learning than specimens from an unstable habitat, a river. The learning abilities of the sticklebacks were not only affected by habitat stability but also by predation pressure. Specimens from the river population, which had longer memory, learned more quickly in a low than high predation risk environment [14]. Mery & Kawecki [15] demonstrated evolution of improved learning ability and better memory in the fruit fly *Drosophila melanogaster*. The fruit flies were selected to associate the taste and/or smell of an oviposition medium with quinine (an aversive chemical cue). After more than 50 generations, the selected flies had a higher learning rate and longer memory duration following classical conditioning [15]. Similarly, Mery *et al*. [16] used classical conditioning, where fruit flies were trained to associate an odor with a mechanical shock, to show that natural polymorphism on a foraging locus affects learning and memory of the flies. Two natural allelic variants were used in the study, one providing for better short-term but worse long-term memory than the other. Flies with the former variant moved more between food patches, which enhanced the speed of learning and favored short-term memory; flies with the latter variant stayed longer within the patches, favoring long-term memory [16].

Long- and short-term diet history may cause both constitutive and proximate (operational) variation in learning ability and performance. This is especially true for diet generalists, like the focal species of our study, *Amblyseius swirskii*, where diet changes and/or diet quality strongly mediate performance in behavior and life history. Cases in point are studies on *Drosophila* sp. For example, Kolss & Kawecki [17] tested the evolutionary trade-off between adaptation to food stress and learning ability in different lines of the fruit flies. Poor food conditions resulted in a decreased learning ability but increased survival rate and faster development. Thus, the physiological adaptations were a direct response to nutritional stress and the reduced learning ability represented a correlated response [17]. Similarly, under a restricted food regime (lower quantity, not quality), fruit flies from a selected low-learning line showed a higher competitive ability than those from a high-learning line [8]. Taken together, these results demonstrate an evolutionary trade-off between adaptation to food stress and learning [8,17]. In many taxa, dietary restriction leads to an extended lifespan, as an adaptation to survive periods of famine, but it is unclear if this affects cognitive aging as well. Burger *et al*. [18] examined the relationship between longevity and the decline of learning ability (memory) with proceeding age under food restriction. Flies grown on a yeast-low diet learned as worse as on a yeast-rich diet, but the line grown on the poor diet lived longer. In contrast, middle-aged flies reared on low-yeast diet had a poorer short-term memory (5 min) than young flies (60 min). Therefore, diet restriction affected the flies’ learning ability but not their cognitive aging [18]. Regarding diet-dependent operational variation of learning, Xia *et al*. [19] discovered that fruit flies reared on two different diets exhibited no and normal learning abilities, respectively. When switching the diets for a few generations, the flies gradually adjusted operant visual learning and memory formation to the new diet. Previously normally learning flies showed a reduced learning ability and poorer memory formation, whereas operant learning and memory formation of the previously non-learning flies returned to a normal level within five generations after transfer to the other diet. Hence, the type of diet profoundly affected the learning behavior of the fruit flies [19].

Proximately, learning may take place via various processes, all of which can roughly be categorized as either associative or non-associative learning [5,20]. Associative learning is a changed response to a stimulus after having learned associating two previously unrelated stimuli or a behavior and a stimulus via reinforcement [21,22]. In contrast, non-associative learning is a changed response to a stimulus following a non-associative experience with a single stimulus, in the absence of any reinforcement [23]. Associative learning comprises processes such as classical and operant conditioning [24,25], non-associative learning comprises sensitization, habituation and imprinting [26–28]. In many contexts, and mainly due to reinforcement, it is assumed that the effects or intensities of learning are commonly, albeit not always and not necessarily, stronger with associative than non-associative processes, but rigorous studies comparing these two learning types in the same learning task are scarce [24]. Different learning processes are not mutually exclusive but are context-dependent and may operate in the same individual simultaneously or sequentially. In general, there is only limited awareness of the fundamental distinction between, and knowledge of the relative importance of, associative and non-associative learning in arthropods. Moreover, this issue aggravates by studies concluding on learning types from loose experimental protocols, which do not allow distinguishing between associative and non-associative processes [29–31].

Research on learning in the focal animals of our study, plant-inhabiting predatory mites of the family Phytoseiidae, is currently gaining momentum, due to their great suitability as model animals in diverse scientific disciplines and their relevance as natural enemies of plant pests. Recent studies provided insights into learning in social [32–34], intraguild (IG) [35], and foraging [28,36–40] contexts. As with many other animals, especially learning early in life is an important determinant of the behavioral and interrelated life historical traits of later life stages [23,41]. Phytoseiid mites have five life stages, egg—larva–protonymph–deutonymph–adult [42], with the larvae and early protonymphs being particularly sensitive for learning in foraging, IG and social contexts [28,32–35,38,40]. For example, regarding foraging, Schausberger *et al*. [28] showed for the predatory mite *Neoseiulus californicus* that memory of thrips contact during the early learning phase, without any feeding experience, persisted into adulthood. Food imprinting during the sensitive phase resulted in adult females having shorter attack latencies and higher predation rates on thrips, as compared to naïve predators. Along the same line, Christiansen *et al*. [40] observed in the focal species of our study, *A*. *swirskii*, well-developed early learning abilities in foraging contexts. Early in life, the predatory mites were allowed experiencing one of two types of prey, that is, difficult-to-grasp (thrips) and easy-to-grasp (spider mites). Memory of early prey experience persisted into adulthood, which was evident in shorter attack latencies on, and higher egg production with, matching prey, that is, the prey type experienced early in life [40].

While the principal learning ability of predatory mites is well documented, the factors responsible for variation in learning ability and performance are virtually unexplored. Accordingly, we addressed this issue in *A*. *swirskii*, which is a broad generalist predatory mite feeding on various mite and insect species [43,44] and non-prey food such as pollen [45,46]. Learning is especially important for diet generalists, because they need to constantly compare, adjust to, and choose among, the available diet options [22,47,48]. Accordingly, several studies showed that generalist feeders with the ability to learn various diet types allocate more time for decision-making than specialist feeders with a restricted range of food and, therefore, reduced learning abilities in foraging contexts [49,50]. *Amblyseius swirskii* originates from the Mediterranean area and is widely used in augmentative biological control of spider mites, thrips and whiteflies in greenhouse crops [43,44,51]. For commercial production, *A*. *swirskii* is reared on a storage pest, the astigmatid mite *Carpoglyphus lactis* [52,53]. Among the range of possible prey types, the western flower thrips, *Frankliniella occidentalis* (Pergande) (Thysanoptera: Thripidae), is considered a difficult-to-grasp prey [40], making learning to improve foraging performance on thrips especially important. We conducted two experiments to, first, find out whether the learning ability of *A*. *swirskii* varies with population origin (commercially mass-reared vs. natural free-living) and rearing diet history (pollen vs. spider mites). We expected that the stable, homogeneous mass-rearing environment with constant super-abundant prey supply selected for decreased learning ability, and that long-term rearing on easy-to-get non-prey food such as pollen compromised the learning performance either constitutively or operationally. Second, we scrutinized whether learning thrips as prey by *A*. *swirskii* [40] takes place via associative and/or non-associative processes. We expected that both learning processes operate in the predators, with associative learning producing stronger effects than non-associative learning.

## Results

### Origin- and diet-related variation of learning (experiment 1)

**Fig 1:**
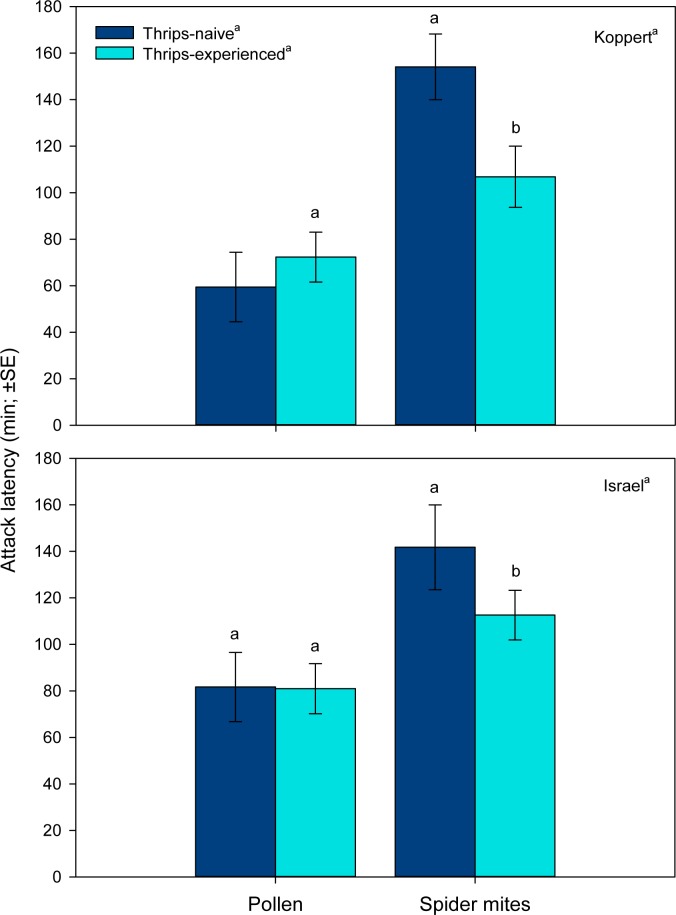
Attack latency (experiment 1). Time elapsed until attack by thrips–naïve and–experienced *Amblyseius swirskii* females, originating from a pollen- or spider mite-reared line of the commercially mass-reared Koppert and the natural free-living Israel population, offered first larvae of thrips *Frankliniella occidentalis* as prey. Thrips-naïve predators were reared on either pollen or spider mites throughout juvenile development, whereas thrips-experienced predators were exposed to thrips during the larval and early protonymphal stage and received then either pollen or spider mites until reaching adulthood. Different superscript letters indicate significant differences between populations, rearing diets and state of thrips experience (GLM; *P* < 0.05). Different letters on top of bars indicate significant differences between thrips-naïve and–experienced predators within the pollen- and spider mite-reared lines (LSD following GLM showing a significant rearing diet*thrips experience interaction; *P* < 0.05).

Rearing diet and thrips experience (marginally significant) but not predator origin as main factors influenced the attack latency of *A*. *swirskii* females (Table 1, Fig 1). Pollen-rearing and thrips experience shortened the attack latencies on thrips as compared to spider mite-rearing and thrips naivity. However, the effect of thrips experience was only evident in predators from spider mite-reared populations, as indicated by the significant interaction term (Table 1, Fig 1).

**Table 1:** Results of generalized linear models (GLMs) on the influence of population origin (commercially mass-reared versus natural free-living), rearing diet (pollen versus spider mites), and thrips experience (yes/no) on the attack latency, total number of eggs, time of first egg and aggregated activity of adult females of the predatory mite *Amblyseius swirskii*. Model selection was based on the lowest QIC values, reached by sequentially removing non-significant interaction terms from the full model.

Dependent variable	Independent variables	Wald*χ*_*1*_^*2*^	*P* value
Attack latency	Population	0.53	0.47
	Rearing diet	33.84	<0.001
	Experience	2.90	0.09
	Rearing diet*experience	5.37	0.02
Total number of eggs	Population	3.95	0.05
	Rearing diet	5.09	0.02
	Experience	0.01	0.91
	Rearing diet*experience	3.65	0.06
Time of first egg	Population	3.82	0.05
	Rearing diet	2.75	0.10
	Experience	0.62	0.42
Aggregated activity	Population	0.04	0.82
	Rearing diet	2.18	0.14
	Experience	0.27	0.60
	Population*rearing diet	11.61	<0.001
	Rearing diet*experience	6.79	<0.001

**Fig 2:**
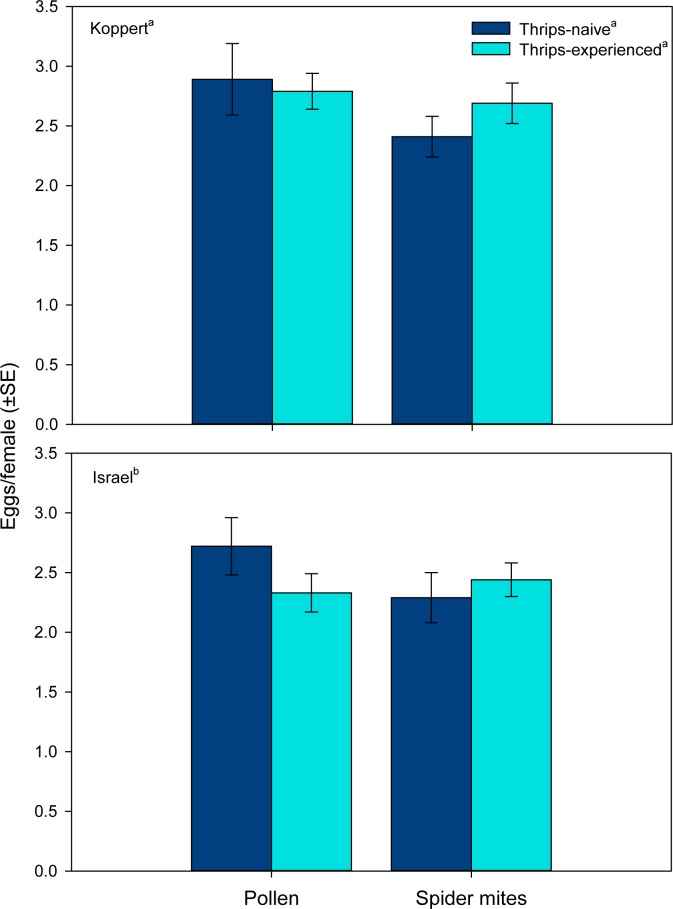
Oviposition (experiment 1). Total number of eggs produced by thrips–naïve and–experienced *Amblyseius swirskii* females, originating from a pollen- or spider mite-reared line of the commercially mass-reared Koppert or the natural free-living Israel population, offered first larvae of thrips *Frankliniella occidentalis* as prey. Thrips-naïve predators were reared on either pollen or spider mites throughout juvenile development, whereas thrips-experienced predators were exposed to thrips during the larval and early protonymphal stage and received then either pollen or spider mites until reaching adulthood. Different superscript letters indicate significant differences between populations, rearing diets and state of thrips experience (GLM; *P* < 0.05).

**Fig 3:**
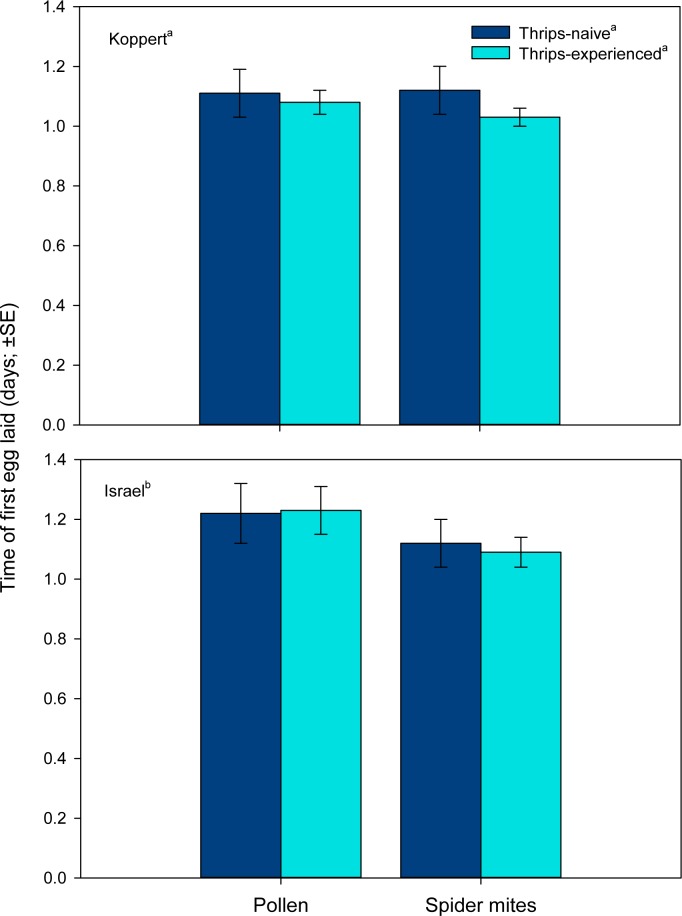
Onset of oviposition (experiment 1). Time of first egg produced by thrips–naïve and–experienced *Amblyseius swirskii* females, originating from a pollen- or spider mite-reared line of the commercially mass-reared Koppert or the natural free-living Israel population, offered first larvae of thrips *Frankliniella occidentalis* as prey. Thrips-naïve predators were reared on either pollen or spider mites throughout juvenile development, whereas thrips-experienced predators were exposed to thrips during the larval and early protonymphal stage and received then either pollen or spider mites until reaching adulthood. Different superscript letters indicate significant differences between populations, rearing diets and state of thrips experience (GLM; *P* < 0.05).

Both predator origin and rearing diet but not thrips experience as main factors influenced the total number of eggs laid by each female. Females derived from the Koppert populations laid more eggs than those from the Israel populations (Table 1, Fig 2). Females from pollen-reared populations produced more eggs than those from spider mite-reared populations. However, the marginally significant interaction between rearing diet and thrips experience indicates that thrips experience increased oviposition in females from the spider mite-reared populations but not in those from the pollen-reared populations (Table 1, Fig 2). The time of the first egg laid was neither affected by rearing diet nor thrips experience, but predator origin had a significant effect (Table 1, Fig 3). Predatory mite females from the Koppert population laid their first egg earlier than females from the Israel population.

**Fig 4:**
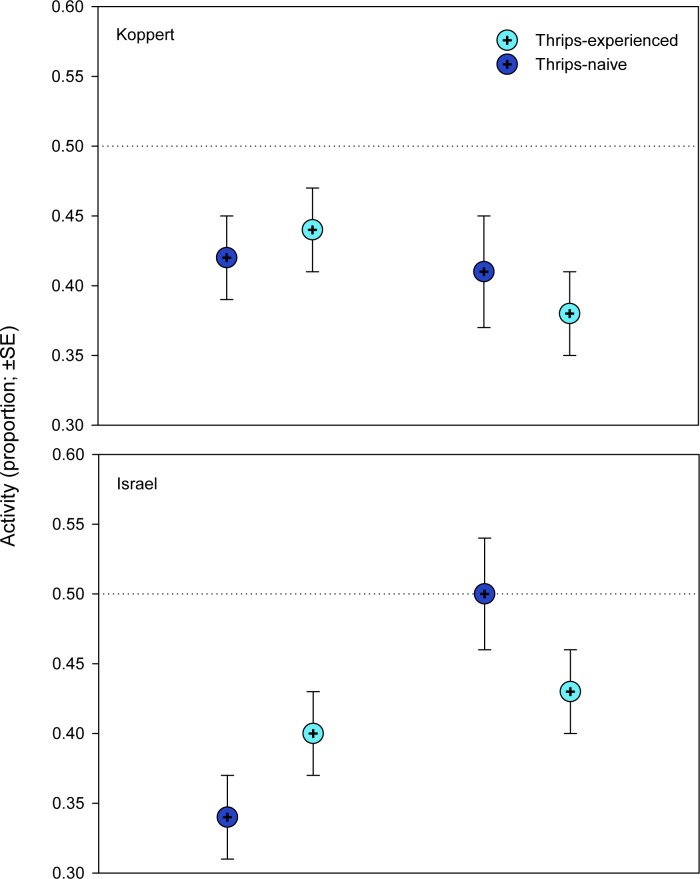
General activity (experiment 1). Proportion of time moving of thrips–naïve and–experienced *Amblyseius swirskii* females, originating from a pollen- or spider mite-reared line of the commercially mass-reared Koppert or the natural free-living Israel population, offered first larvae of thrips *Frankliniella occidentalis* as prey. Thrips-naïve predators were reared on either pollen or spider mites throughout juvenile development, whereas thrips-experienced predators were exposed to thrips during the larval and early protonymphal stage and received then either pollen or spider mites until reaching adulthood. GLM revealed significant population*rearing diet and rearing diet*thrips experience interactions (*P* < 0.001).

None of the main factors influenced the activity of the predatory mite females. However, the interactions between predator origin and rearing diet and thrips experience and rearing diet, respectively, had a highly significant influence on activity. Within the spider mite-reared populations, thrips-experienced females were less active than thrips-naïve females, whereas the reverse was true within pollen-reared populations (Table 1, Fig 4).

**Fig 5:**
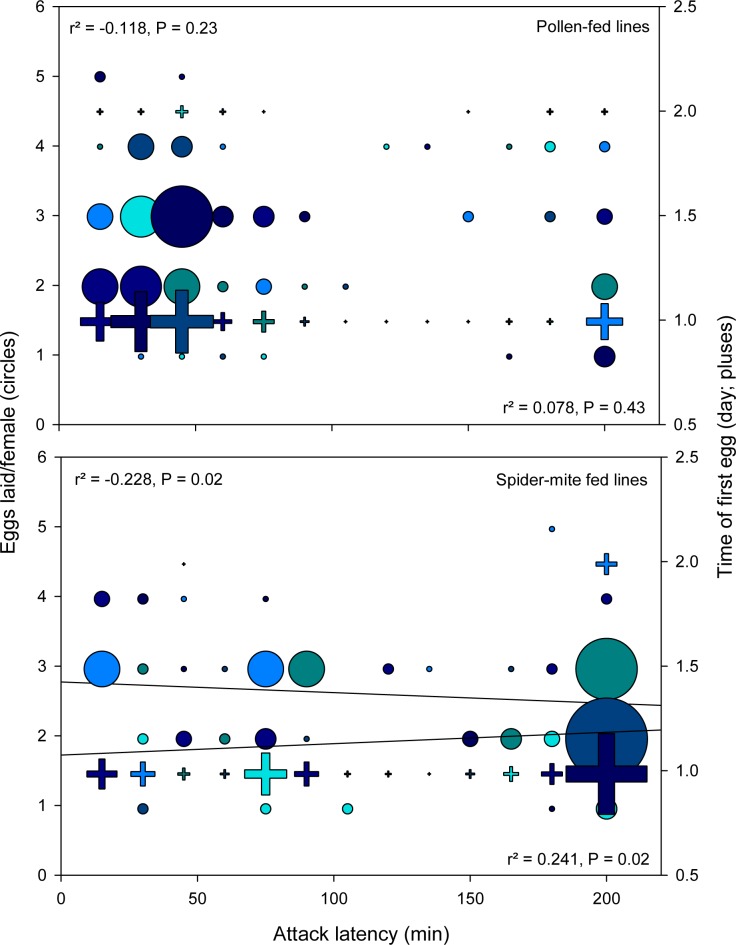
Correlation analyses (experiment 1). Total number of eggs (circles) and time of first egg (pluses) regressed on attack latency of *Amblyseius swirskii* females from the pollen- and spider mite-reared lines (Koppert and Israel populations lumped). Symbol size is proportional to sample size. Statistical results refer to bivariate linear regressions.

Bivariate correlation analyses revealed a significant negative correlation between the attack latencies and total number of eggs and a significant positive correlation between the attack latencies and time of first egg in females from spider mite-reared populations but not in females from pollen-reared populations (Fig 5).

### Associative vs. non-associative learning (experiment 2)

**Fig 6:**
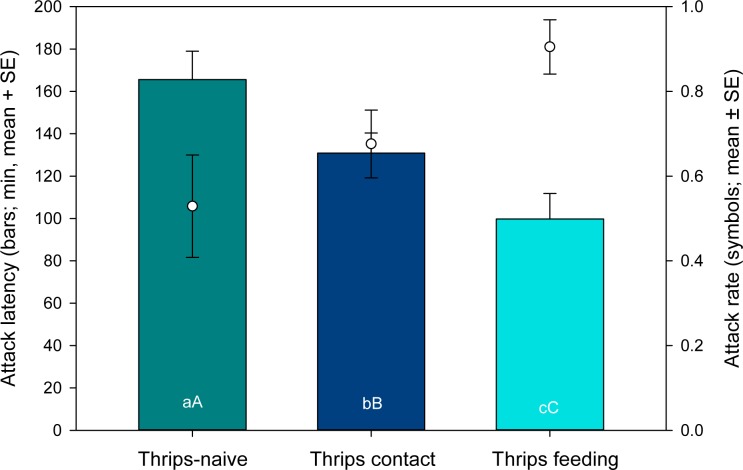
Non-associative versus associative learning (experiment 2). Attack latency and attack likelihood of thrips-naïve and -experienced *Amblyseius swirskii* females offered first larvae of thrips *Frankliniella occidentalis* as prey. Thrips-naïve predators were reared on spider mites throughout juvenile development, whereas thrips-experienced predators were allowed to either contact thrips (non-associative learning) or feed on thrips (associative learning) during the larval and early protonymphal stage and received then spider mites until reaching adulthood. Different lower and upper case letters inside bars indicate significant differences in attack latency and attack rate among thrips-naïve and–experienced (contact or feeding) predators (GLM; *P* < 0.05).

Type of thrips experience had a significant influence on both, the attack latency (GLM: Wald *χ*^*2*^_2_ = 13.361, *P* = 0.001), and the attack likelihood (Wald *χ*^*2*^_2_ = 6.572, *P* = 0.037) (Fig 6). Associative learners attacked thrips more likely and earlier than non-associative learners than naïve predators.

**Fig 7:**
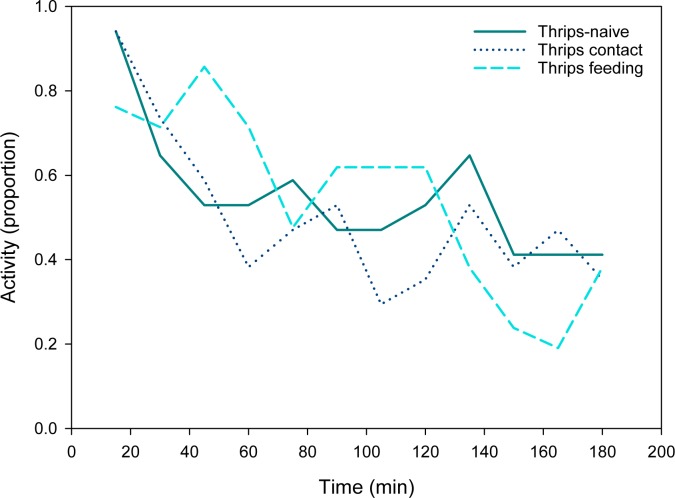
General activity (experiment 2). Proportion of time moving of thrips-naïve and -experienced *Amblyseius swirskii* females offered first larvae of thrips *Frankliniella occidentalis* as prey. Thrips-naïve predators were reared on spider mites throughout juvenile development, whereas thrips-experienced predators were allowed to either contact thrips (non-associative learning) or feed on thrips (associative learning) during the larval and early protonymphal stage and received then spider mites until reaching adulthood.

Type of thrips experience as main factor had no effect on activity (moving or stationary; GEE: Wald *χ*^*2*^_2_ = 0.860, *P* = 0.65) but had a highly significant influence over time (Wald *χ*^*2*^_33_ = 85.342, *P* < 0.001). All females reduced their activity in the course of the experiment but associative learners were more active within the first 120 min and less active later on than non-associative learners and naïve predatory mite females (Fig 7).

## Discussion

Our study provides evidence for constitutive and/or operational variation, caused by diet history (pollen vs. spider mites), and operational variation, caused by the type of experience (associative vs. non-associative), of the learning ability and performance of the predatory mite *Amblyseius swirskii*. Depending on the influence of the above factors and their interactions, larval experience with the Western flower thrips *Frankliniella occidentalis* altered the behavior of the predatory mites as adult females or not. Population origin (mass-reared vs. natural) did not cause variation in learning ability but was responsible for constitutive variation in egg production.

### Origin- and diet-related variation of learning

The first experiment corroborates the findings by Christiansen *et al*. [40] that *A*. *swirskii* can learn during an early sensitive phase and memorize as adults (after two molting events) the difficult-to-grasp prey thrips. Rearing diet, spider mites vs. pollen, but not population origin, commercially mass-reared vs. natural free-living, affected the learning performance. Regarding learning, many animals have an especially sensitive phase immediately after birth or hatching, albeit some may already learn in the pre-natal phase [39,54]. In phytoseiid mites, the post-natal sensitive stages are the larvae and early protonymphs [28], which last, at 25°C, around 3 days after hatching [42]. Learning by larvae/early protonymphs was most evident in shortened attack latencies by adult females from the spider-mite reared lines. Predatory mites are generally time-limited in their foraging behavior, making the searching and handling times, which include the times needed to recognize, accept and attack prey, highly relevant for optimizing their foraging efforts [38]. Shortened attack latencies increase prey profitability, i.e., the net energy gain per prey item, and should consequently increase the reproductive success [55]. Optimized foraging time budget was also reflected in general activity. Within the spider mite-reared lines, thrips-experienced females were less active than naïve females, whereas the reverse was true within pollen-reared lines. Under the circumstances tested, reduced general activity is adaptive because saving energy and allowing investing more energy, and spending more time, for feeding and producing eggs, enhancing the reproductive success. Strikingly, shorter attack latencies correlated with a higher total number of eggs in females from the spider mite-reared but not pollen-reared lines, indicating the adaptive significance of learning (pollen-reared individuals did not learn).

Thrips-experienced females from both lines reared on spider mites attacked thrips faster than did thrips-naïve females. In contrast, larval thrips experience had no effect on the attack latency of females from the two pollen-reared lines. However, pollen-reared predators attacked thrips earlier, no matter whether thrips-experienced or not, and produced more eggs than spider mite-reared predators. Shorter attack latencies of previously pollen-fed than spider mite-fed predators might reflect a stronger propensity to ingest animal prey for obtaining a balanced diet. *A*. *swirskii* has a superior life history performance on some mixtures of plant and animal diets, such as pollen and thrips, than on single diets [56]. Lack of learning by pollen-reared predators may be either a short-term consequence of nutritional deficiency or shifted nutrient/energy allocation or a long-term consequence due to laboratory selection for loss of learning ability. Nutritional deficiency may lead to lacking or reduced learning performance and is known from both invertebrates [19,57] and vertebrates [58–60]. Shifted nutrient/energy allocation may have led to short-term trade-offs between learning and egg production. If one of the two short-term options was the case in our experiments, the learning ability should be restored after returning to a high quality, nutrient-rich diet. Another possible explanation for the lost learning ability in pollen-reared predators is laboratory selection. Maintenance of learning ability, in the case of *A*. *swirskii* for enhancing predation, is physiologically costly [61] and not needed in an environment that is persistently full of easy-to-get pollen but absence of living prey. Before conducting the experiments, we reared the two pollen lines for approximately 11 months on pollen, which are >30 generations assuming a generation time of ~10 days. Assuming a trade-off between life history performance and maintenance of learning ability [61], specimens producing more eggs but lacking learning ability may have become dominant over time, gradually displacing specimens maintaining learning ability at the expense of lower egg production (see [17] for an analogous phenomenon in *D*. *melanogaster*). Similar phenomena were found in fruit flies *Drosophila* sp. [8,9], one of the most widely used model organism in learning studies, and butterflies [62]. Mery & Kawecki [8] conducted experiments with two different outbred lines, a high-learning line and a low-learning line originating from the same stock population, that is, the same genepool. Under food limitation, the high-learning line was better able to learn an aversive smell and taste of a substrate medium but had poorer larval competitive abilities [8]. In another experiment, Mery & Kawecki [9] exposed the two lines to different oviposition media in 12 consecutive 48 h cycles under mild nutritional stress. The conditioned high-learning line laid fewer eggs over time than the low-learning line. Furthermore, along the consecutive cycles of conditioning, the high-learning line revealed an apparent trade-off between learning score and egg production [9]. Snell-Rood *et al*. [62] observed in the small white butterfly, *Pieris rapae*, that individuals from a better learning line paid the costs of producing fewer eggs than those from a poorer learning line.

Since individuals from the commercially mass-reared and the natural free-living populations did not fundamentally differ in learning performance, we conclude that long-term commercial mass-rearing did not constitutively compromise the learning ability of the predatory mite *A*. *swirskii*. *Amblyseius swirskii* is widely used in augmentative biological control and commercially reared on a storage pest, the astigmatid mite *C*. *lactis*. In the commercial mass-rearing facilities the predators are exclusively exposed to a single prey type, which is super-abundantly present. We assumed that learning is not that important in such an environment and might thus gradually degrade over time, due to the benefit-cost trade-offs of learning. However, there was no indication whatsoever for a negative effect of long-term mass rearing on learning ability of *A*. *swirskii*. At the very beginning of our study, both the Koppert and Israel populations were each split into two lines, one reared on spider mites and the other on pollen. The spider-mite reared lines of both populations had similarly well-developed learning abilities, suggesting that commercial, long-term mass-rearing on one type of prey either did not select for reduction or loss of learning ability or that the producers refresh the gene-pool of the rearing from time to time by adding wild specimens. In contrast to learning ability, we observed a constitutive population difference in the reproductive potential of the commercially mass-reared and the natural free-living populations; females from the Koppert population laid their first egg earlier and in total more eggs than females from the Israel population. Possible explanations are that the mass-rearing conditions set the stage for selecting individuals with high reproductive potential and/or the producers selectively founded, or refreshed, the mass-rearing with reproductively high-performing individuals.

### Associative vs. non-associative learning

The second experiment emphasizes the difference in learning intensity produced by associative and non-associative prey experiences. Type of experience influenced both, the attack likelihood and the attack latency on thrips. Associative learners (feeding on thrips early in life) attacked thrips more likely and more quickly than non-associative learners (contact with thrips early in life) than thrips-naïve individuals. Moreover, type of thrips experience influenced the time-dependent activity of the predators. Associative learners were initially, upon presentation of thrips, more active but, towards the end of the 3 h observation period, less active than non-associative learners and thrips-naïve individuals.

There exist numerous studies demonstrating either non-associative or associative learning, yet only a few rigorous experimental designs highlighted the relative importance of, and distinction between, these two learning categories in one and the same species and learning task [24,63,64]. At the molecular level, Pereira & van der Kooy [63] revealed an odorant-specific ability to employ non-associative and associative learning in the nematode *C*. *elegans*. Most strikingly, they found that, although the same AWC sensory neurons were involved, gene activity differed between non-associative and associative learning. Mogensen *et al*. [64] revealed through imipramine injections that serotonergic and anticholinergic receptors in the neural system were involved in non-associative and associative learning. At the behavioral level, Kaiser *et al*. [24] sensitized the fruit fly parasitoid *Leptopilina boulardi* during oviposition or classically conditioned them to an odor, evaluating their abilities for short- and long-term memory in host location. Sensitization and one time associative experiences led to short term memory, whereas repeated associative experiences resulted in both short- and long-term memory. In the associative learning paradigm of our experiment, *A*. *swirskii* was presented chemo- and mechano-sensory cues through contact with prey and allowed associating these cues used for prey recognition with gustatory cues through prey ingestion and satiation. The reinforcing cues were missing in the non-associative learning paradigm [23], where the predators could only perceive chemo- and mechano-sensory prey cues by contact but did not attack and ingest prey. Both non-associative and associative learning resulted in long-term memory, lasting for several days throughout juvenile development. Similarly, in another predatory mite, *N*. *californicus*, memory following non-associative prey experience was shown to persist for several days after reaching adulthood. While the experiments presented here and previous studies [28,40] did not find any indication that non-associative and associative experiences in early life differ in the length of memory retention in predatory mites, they show that the two types of learning differ in the intensity of the produced learning effect. Whether the two types differ at the molecular level regarding gene activity, or associative learning just activates additional genes to those already activated by non-associative learning remains to be assessed in future studies. In any case, our experiments corroborate previous findings that mere non-associative experiences in early life have unusually strong and persistent effects in predatory mites in foraging and social contexts [28,32–34,40], clearly pointing at the existence of a highly sensitive time window for learning in early life.

## Conclusions

Our study provides evidence that thrips experience by *A*. *swirskii* females early in life alters their behavior towards this prey as adults, and is one of a few studies rigorously comparing the effects of associative and non-associative learning in one and the same learning task. Rearing diet had a decisive influence on learning performance, but our experimental design was not apt to elucidate the mechanisms of the compromised learning ability of predatory mites reared on pollen. We argue that the observed behavioral changes indicate a reduced learning ability as a consequence of either nutritional deficiency, nutrient allocation shift or laboratory selection, but further research is needed to shed light on these matters. The finding that population origin, long-term mass rearing on a factitious prey, did not compromise learning, as compared to the population collected in the field, is relevant for the use of *A*. *swirskii* in biological control. The presence of a specific prey or their cues during rearing may increase the efficacy of the biological control agents, if these are then better able to recognize, handle and attack this prey in the crop. *Amblyseius swirskii* is widely used in biological control of thrips [43,44,51], but is commonly mass-reared on astigmatic mites and thus does never experience thrips before release in the greenhouse crop [52,53]. As previously suggested [28,40], evaluating ways to prime or condition biological control agents before their release in the field, and thus, enhance their efficacy against a specific pest, represents exciting future research topics.

## Materials and Methods

### Predator origins and predator and prey rearing

The four *Amblyseius swirskii* lines used in the experiments derived from two origins, each reared in two different ways. Two lines were founded with specimens from a commercially mass-reared population obtained from Koppert B.V. (The Netherlands) while the other two lines were founded with free-living specimens collected on citrus trees in Israel. No specific permission was required to collect the mites on the citrus trees because those trees were located on public grounds and *A*. *swirskii* is not an endangered or protected species. In the laboratory, all four lines were reared on separate artificial arenas for about 11 months (~30 generations) before conducting the experiments. One line of each population origin was reared with cattail pollen *Typha angustifolia* (Nutrimite; Biobest, Belgium), and the other line with two-spotted spider mites, *Tetranychus urticae* (Tetranychidae). Each rearing arena consisted of an acrylic plate (200 x 200 mm) placed on top of a water-saturated foam cube in a plastic box half-filled with tap water. Wet tissue paper was wrapped around the edges to establish a border between the acrylic plate and the surrounding water, and to prevent the predatory mites from escaping. Additionally, cotton wool fibres under coverslips served as shelters and oviposition sites for the predatory mites. Pollen was dusted onto arenas twice per week. *Tetranychus urticae* was reared on whole common bean plants *Phaseolus vulgaris* grown at room temperature 23 ± 2°C and 16:8 h L:D photoperiod. The spider mites were brushed from infested leaves onto glass plates, using a mite brushing machine (BioQuip®, USA), and then from glass plates onto the rearing arenas. Depending on the population origin (KO for Koppert; IL for Israel) and rearing food (PO for pollen; SM for spider mites), the henceforth-used acronyms of the four lines are KO-PO, KO-SM, IL-PO and IL-SM.

Prey used in the experiments were Western flower thrips *Frankliniella occidentalis* (Thripidae) and two-spotted spider mites *T*. *urticae*. *Frankliniella occidentalis* was reared on detached primary leaves of common bean *P*. *vulgaris* placed upside down on a 1% agar solution in a closed petri dish (140 mm Ø, 20 mm height). The lid of the petri dish had a hole (10 mm Ø) covered with gauze for ventilation. Only first instar larvae were used as prey in the experiments. To obtain first instar larvae, adult thrips females were randomly taken from the stock population, reared on whole green beans inside glass jars, and placed on detached bean leaves inside petri dishes for oviposition. After 24 h, the females were removed and after another ~70 to 80 h the first instar larvae hatched [40].

Predator rearing arenas, thrips rearing units and experimental cages were kept in climate chambers at 25±1°C, 65±5% relative humidity and 16:8 h L:D photoperiod.

### Experimental procedures

To obtain similarly-aged eggs giving rise to experimental individuals, predatory mite females were randomly taken from the stock population and placed for oviposition on detached bean leaf arenas and provided with spider mite prey. Each bean leaf arena (50 x 50 mm) consisted of a trifoliate bean leaf placed upside down on a water-saturated foam cube in a plastic box half-filled with tap water. Wet tissue paper was wrapped around the edges of the leaves to establish a border between the bean leaf and the water, and prevent the predatory mites and their prey from escaping. After 24 h, the eggs were collected using a fine moistened brush and placed singly into acrylic cages (15 mm Ø, 3 mm height). The cages were closed at the bottom with gauze and covered on the upper side with a microscope slide. To warrant elevated humidity inside, the cages were stored on a grid above tap water in an open plastic box [65].

### Origin- and diet-related variation of learning (experiment 1)

The first experiment aimed at assessing the influence of predator origin (commercially mass-reared KO vs. natural free-living IL) and rearing diet (pollen vs. spider mites) on early learning thrips as prey. To this end, young predators were exposed in the larval and early protonymphal stage to thrips larvae or not. Two predator origins, two rearing diets and thrips experience (yes/no) resulted in eight treatments (Table 2).

**Table 2:** Predator origin, rearing diet and diet during the experimental phases in experiment 1. Female offspring from mothers derived from the spider mite- or pollen-reared lines of the Koppert and Israel populations of *Amblyseius swirskii* were, in the larval and early protonymphal stage (experience phase), exposed to thrips larvae, *Frankliniella occidentalis*, or not (naïve), then fed on the rearing diet of the line they came from until reaching adulthood (consolidation phase) and, after mating, tested for predation on thrips (behavioral assay).

Population	Line		Diet during experimental phase			Learning status
	Acronym	Rearing diet	Experience	Consolidation	Behavioral assay	
Koppert	KO-PO	Pollen	Pollen	Pollen	Thrips	Naive
			Thrips	Pollen	Thrips	Experienced
	KO-SM	Spider mites	Spider mites	Spider mites	Thrips	Naive
			Thrips	Spider mites	Thrips	Experienced
Israel	IL-PO	Pollen	Pollen	Pollen	Thrips	Naive
			Thrips	Pollen	Thrips	Experienced
	IL-SM	Spider mites	Spider mites	Spider mites	Thrips	Naive
			Thrips	Spider mites	Thrips	Experienced

The experimental procedure consisted of three phases: experience, consolidation and behavioral assay (Table 2). The experience phase was started by placing age-synchronized *A*. *swirskii* eggs singly into acrylic cages that contained either their rearing diet (pollen or 10 eggs plus 5 juvenile spider mites) or three first instar larvae of *F*. *occidentalis*, always together with a 2 x 2 mm piece of bean leaf. The bean leaf piece was added to the cages to reduce predator egg killing by thrips [66]. During all experimental phases, specimens fed with pollen were additionally provided free water (see [65] for details of water provisioning inside cages). During the experience phase the cages were checked twice per day in 6 h intervals for determining the developmental progress of the predators. The experience phase ended as soon as the predatory mites had molted to protonymphs. Thus, in their larval and early protonymphal stage the predatory mites experienced either the rearing diet of the population they came from, either pollen or spider mites (for naïve), or thrips (for experienced). After reaching the protonymphal stage, which happened ~1.5 to 2 days after hatching, the second phase, dubbed consolidation, started. Each thrips-naïve and -experienced protonymph was transferred, using a fine moistened marten’s hair brush, to a new cage and provided with the rearing diet of the population they came from, i.e. either pollen or spider mites. During the consolidation phase, the cages were checked once per day to monitor the predators’ developmental progress from protonymph to deutonymph to adult, which lasted ~4 to 5 days. Adult females were transferred to a new cage, provided with the rearing diet of the population they came from, and an adult male, randomly taken from the same population as the female came from, was added. After 24 h the males were removed and the females were ready for the third phase, i.e. the behavioral assay. For the behavioral assay, each female was singly placed into a cage previously loaded with five first instar larvae of *F*. *occidentalis*. The cages were monitored every 15 min for three hours and then again after 24, 48, 72 and 96 h to determine the occurrence and number of dead thrips (indicative of the time until the first successful attack of the predatory mites, i.e. their attack latency), the predator activity (stationary/moving) and the number of eggs produced. Predator eggs were daily removed from the cages. Activity and oviposition parameters (time of first egg and number of eggs) were recorded as potential indicators of energy allocation shifts and the adaptive significance of learning. Each of the eight treatments was replicated 17 to 39 times. Females not laying a single egg were discarded from analyses, assuming fertilization failure.

### Associative vs. non-associative learning (experiment 2)

The second experiment aimed at discriminating between non-associative and associative learning processes. In this experiment, we only used the spider mite–reared population from Israel (IL-SM).

We compared three groups of predators, associative learners, non-associative learners and naïve individuals. The three phases of the experimental procedure were in principle the same as those in the first experiment (Table 3). In the experience phase, during the larval and early protonymphal stage, the predators received either spider mites (naive), or three living thrips larvae (for non-associative learning), or two living and one dead, manually killed using a needle, thrips larvae (for associative learning) (Table 3). The non-associative and associative learning treatments differed in that in the former treatment thrips were only contacted but not killed and fed upon by the predators, whereas in the latter treatment the predators could contact and easily feed on thrips. Replicates in the non-associative learning treatment with occurrences of dead thrips were discarded. Upon reaching the protonymphal stage, for the consolidation phase the predators were transferred to new cages and provided with spider mites until reaching adulthood. Adult females were provided with a male and, as in experiment 1, on the next day subjected to the behavioral assay. The protocol of the behavioral assay was the same as in experiment 1. Each treatment was replicated 17 to 34 times.

**Table 3:** Predator origin, rearing diet and diet during the experimental phases in experiment 2. Female offspring from mothers derived from the spider mite-reared line of the Israel population of *Amblyseius swirskii* (IL-SM) were, in the larval and early protonymphal stage (experience phase), exposed to thrips larvae, *Frankliniella occidentalis*, or not (naïve), then fed on spider mites until reaching adulthood (consolidation phase) and, after mating, tested for predation on thrips (behavioral assay). In the experience phase, the predators were allowed either to only contact thrips (non-associative learning) or to contact and feed on thrips (associative learning) or to contact and feed on spider mites (thrips-naïve).

Population	Line		Diet during experimental phase			Learning status
	Acronym	Rearing diet	Experience	Consolidation	Behavioral assay	
Israel	IL-SM	Spider mites	Thrips feeding	Spider mites	Thrips	Associative
			Thrips contact	Spider mites	Thrips	Non-associative
			Spider mites	Spider mites	Thrips	Naive

### Statistical analyses

Statistical analysis was carried out using IBM SPSS 21 (IBM Corp., USA). In experiment 1, we used generalized linear models (GLMs) to analyze the influence of population origin, rearing diet and early thrips experience on the attack latency (normal distribution, identity link), aggregated activity (binomial distribution, logit link, counts of events), time of first egg (day 1 or 2; binomial distribution, logit link), and total number of eggs (Poisson distribution, log link). For analysis of attack latency, we used a stopping time of 200 min for predators attacking after the initial 180 min observation period. Before analysis of activity, the repeated observations were aggregated into one value for each individual, i.e. the number of times the predators were observed moving out of all 16 observations. We used bivariate linear correlations to examine the relationship between attack latency and total number of eggs and time of first egg separately for pollen- and spider mite-fed predators.

In experiment 2, we used generalized linear models (GLMs) to analyze the influence of type of experience (no, contact or feeding) on the attack latency (normal distribution, identity link) and attack likelihood within the observation period (binomial distribution, probit link). To analyze the activity (moving/stationary) of the predatory mite females as influenced by type of experience and experience nested in time, generalized estimating equations (GEE; binomial distribution with probit link; autocorrelation structure between observation points) were conducted.

## Supporting Information

S1 Table Raw data of the experiments. (XLS)
