# Correction: Constitutive and Operational Variation of Learning in Foraging Predatory Mites

**DOI:** 10.1371/journal.pone.0171450

**Published:** 2017-01-30

**Authors:** Michael Seiter, Peter Schausberger

The x-axis is missing from Fig 4. Please see the complete, corrected Fig 4 here.

**Fig 4:**
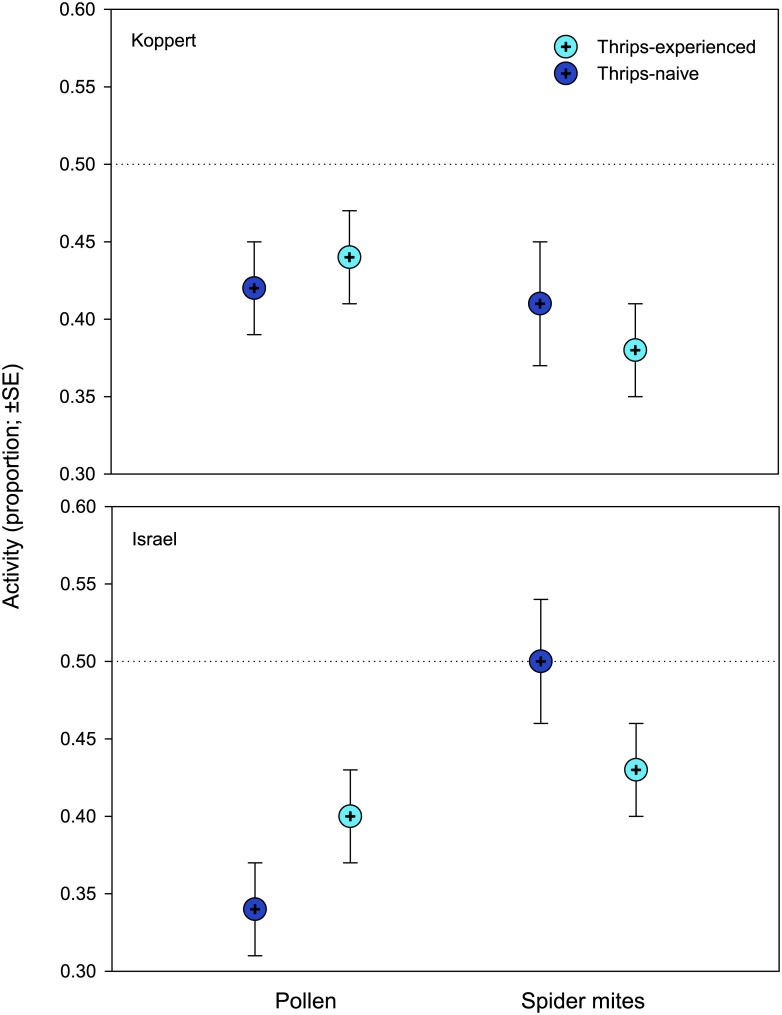
General activity (experiment 1). Proportion of time moving of thrips–naïve and–experienced *Amblyseius swirskii* females, originating from a pollen- or spider mite-reared line of the commercially mass-reared Koppert or the natural free-living Israel population, offered first larvae of thrips *Frankliniella occidentalis* as prey. Thrips-naïve predators were reared on either pollen or spider mites throughout juvenile development, whereas thrips-experienced predators were exposed to thrips during the larval and early protonymphal stage and received then either pollen or spider mites until reaching adulthood. GLM revealed significant population*rearing diet and rearing diet*thrips experience interactions (*P* < 0.001).
